# High-Fat Diet Aggravates the Intestinal Barrier Injury via TLR4-RIP3 Pathway in a Rat Model of Severe Acute Pancreatitis

**DOI:** 10.1155/2019/2512687

**Published:** 2019-12-17

**Authors:** Ying-ru Su, Yu-pu Hong, Fang-chao Mei, Chen-yang Wang, Man Li, Yu Zhou, Kai-liang Zhao, Jia Yu, Wei-xing Wang

**Affiliations:** ^1^Department of General Surgery, Renmin Hospital of Wuhan University, Wuhan, Hubei, China; ^2^Hubei Key Laboratory of Digestive System Disease, Wuhan, Hubei, China

## Abstract

**Objective:**

For patients with severe acute pancreatitis (SAP), a high body mass index (BMI) increases the possibility of infection derived from the intestine. In this study, we evaluate whether TAK242 can alleviate severe acute pancreatitis-associated injury of intestinal barrier in high-fat diet-fed rats.

**Methods:**

A SAP model was established by retrograde injection of 5% sodium taurocholate into the biliary-pancreatic duct. Thirty Sprague-Dawley (SD) adult rats were randomly divided into five groups: standard rat chow (SRC) normal (SN), SRC SAP (SAP), high-fat diet normal (HN), HFD SAP (HSAP), and TLR4 inhibitor pretreatment HFD SAP (HAPT) groups. Intraperitoneal injection of 3 mg/kg TAK242 was administered 30 minutes before SAP model establishment in the HAPT group. Rats were sacrificed 12 hours after SAP modeling, followed by blood and pancreatic and distal ileum tissue collection for further analyses. Changes in the pathology responses of the rats in each group were assessed.

**Result:**

Analyses of serum amylase, lipase, cholesterol, triglyceride, IL-1*β*, IL-6, DAO, and serum endotoxin as well as tight junction protein expression including zonula occluden-1 and occludin indicated that high-fat diet aggravated SAP-induced intestinal barrier injury via increasing inflammatory response. In addition, the level of necroptosis was significantly higher in the SAP group compared with the SN group while the HSAP group exhibited more necroptosis compared with the SAP group, indicating the important role of necroptosis in pancreatitis-associated gut injury and illustrating that high-fat diet aggravated necroptosis of the ileum. Pretreatment with TLR4 inhibitor significantly alleviated inflammatory response and reduced necroptosis and level of oxidative stress while improving intestinal barrier function.

**Conclusion:**

High-fat diet aggravated SAP-induced intestinal barrier injury via inflammatory reactions, necroptosis, and oxidative stress. Inhibition of TLR4 by TAK242 reduced inflammation, alleviated necroptosis, and lowered the level of oxidative stress and then protected the intestinal barrier dysfunction from SAP in high-fat diet-fed rats.

## 1. Introduction

Acute pancreatitis (AP) is one of the most common acute abdominal diseases in clinic. AP is usually a mild and self-limiting disease, and about 20% of cases may develop into severe acute pancreatitis (SAP) leading to early systemic inflammatory response syndrome (SIRS) and even multiple organ failure syndrome (MODS) [1].

Obesity has become more common as people's living standards improve. Nearly 30% of the world's population is overweight or consumes high-fat diet. And if current trends continue, approximately 50% of the world's population is projected to consume high-fat diet by 2050 [2]. Not only considered as a risk factor for AP, obesity is also associated with multiple complications following AP [3]. Toll-like receptor 4 (TLR4) is known as a component of immune system pattern recognition receptors (PRRs) and plays a crucial role as a trigger of metabolic inflammation and insulin resistance during obesity [4].

Toll-like receptors belong to a crossmembrane glycoprotein receptor family, which can recognize family that can recognize pathogen-associated molecular patterns (PAMPs) [5]. TLR4 was one of the earliest found and most studied members in the toll-like family. TLR4 recognizes PAMPs and damage-associated molecular pattern (DAMPs) to activate nuclear factor-kappa B (NF-*κ*B) by cascade amplification of external signals, promoting cytokine production and release [6]. In addition, TLR4 plays an important role in facilitating necroptosis through inflammatory factors such as tumor necrosis factor-*α* (TNF-*α*).

Necroptosis is a form of programmed cell death acknowledged in recent years and depends on the activity of the receptor-interacting protein (RIP) kinase family, which could be triggered by TNF-*α* in a classical pathway [7]. Necroptosis has been shown to be mediated by the kinases RIP1 and RIP3. Epithelial cell death is a hallmark of intestinal inflammation and has been discussed as a pathogenic mechanism driving inflammatory bowel disease (IBD) in humans [8]. Studies found that epithelial cell death was induced by TNF-*α*, accompanied by increased expression of RIP3, and could be inhibited upon blockade of necroptosis [9–11]. Intestinal epithelial homeostasis is maintained by a strict equilibrium between cell proliferation in the crypt and cell shedding from the villus tip [12].

Intestinal barrier consists of four parts: mechanical barrier, chemical barrier, immune barrier, and biological barrier [13]. The change of tight junction among intestinal epithelial cells plays a significant role in increasing of intestinal permeability in SAP [14]. However, the role of toll-like receptors in the regulation of intestinal integrity is poorly understood and the role of necroptosis in cell shedding and barrier loss has not been reported. Consequently, the aim of this study is to investigate whether TAK242 could decrease the level of necroptosis and production of inflammatory factors in HFD-fed rats with SAP to alleviate the intestinal barrier injury.

## 2. Materials and Methods

### 2.1. Material and Reagents

The high-fat diet (HFD, D12492) chow was purchased from Beijing Huafukang Bioscience; standard rat chow (SRC) was offered by the experimental animal center of Wuhan University Renmin Hospital. Rat ELISA kits of interleukin- (IL-) 1*β* (E-EL-R0012c) and IL-6 (E-EL-R0015c) were purchased from Elabscience (Wuhan, China). Diamine oxidase (DAO) assay kit (A088-1) and endotoxin assay kit (E039-1-1), reduced form of glutathione (GSH) assay kit (A006-2-1) and maleic dialdehyde (MDA) assay kit (A003-1-2), and superoxide dismutase (SOD) assay kit (A001-2-2) were purchased from Nanjing Jiancheng Bioengineering Institute (Nanjing, China). Zonula occluden-1 (Zo-1) (21773-1-AP) and occludin (27260-1-AP) antibodies were purchased from Proteintech (Wuhan, China); RIP3 (ab56164) and TNF-*α* (ab6671) antibodies were purchased from Abcam (Cambridge, UK); TLR4 (GB11519), NF-*κ*B P65 (GB11142), and *β*-actin (GB11001) antibodies were purchased from Servicebio (Wuhan, China). TAK242 (HY-11109), an inhibitor of TLR4, was purchased from MCE (Shanghai, China).

### 2.2. Animals and Modeling

**Figure 1:**
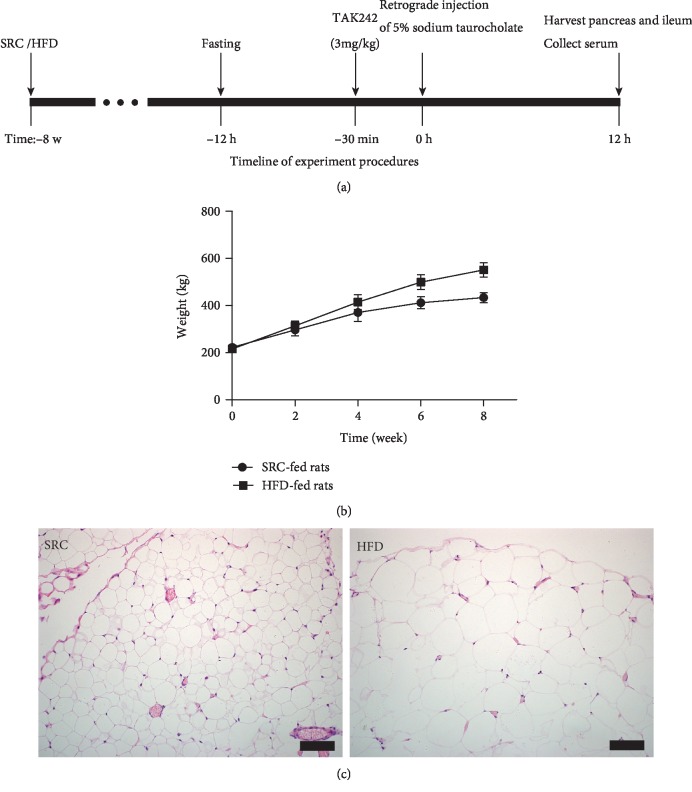
HFD-fed rats showed a higher body weight and bigger volume of adipocytes. (a) Timeline of experiment procedures. (b) HFD-fed rats showed a higher body weight compared with SRC-fed rats. (c) HFD-fed rats showed bigger volume of adipocytes compared with SRC-fed rats.

Thirty Sprague-Dawley (SD) adult rats (200–220 g) were bought from the experimental animals Hunan SJA Laboratory Animal Company (Changsha, Hunan, China). Those SD adult rats were adapted for 3 days. The indoor temperature was maintained at 20-25°C, the humidity was 35%-45%, and the rats were exposed to light for 12 hours. The rats were free to drink and chow. Rats were randomly divided into five groups: SRC normal (SN, *n* = 6), SRC SAP (SAP, *n* = 6), HFD normal (HN, *n* = 6), HFD SAP (HSAP, *n* = 6), and TAK242 (inhibitor of toll like receptor-4) pretreatment for HFD SAP (HAPT, *n* = 6) groups. After 8 weeks feeding on SRC or the HFD, the rats were operated for SAP modeling and euthanized at 12 h after the operation. The SAP model was established by retrograde injection of 5% sodium taurocholate into the bile and pancreatic duct. Intraperitoneal injection of 3 mg/kg TAK242 was administered 30 minutes before HFD SAP model establishment in the HAPT group [15]. After closure, the rats were supplemented with saline subcutaneously (20 ml/kg weight) for fluid loss (Figure 1(a)).

### 2.3. Sample Collection

Rats were sacrificed and blood samples were obtained from the inferior vena cava. Blood samples were centrifuged for 10 min at 4000×g, and serum were conserved at −80°C. Pancreases and distal ileum tissues were immediately frozen in liquid nitrogen and conserved at −80°C for further analyses.

### 2.4. Serum Assay

The activities of amylase (AMY) and lipase (LIP) and levels of total cholesterol (TC) and triglyceride (TG) in serum were detected by the Automatic Biochemistry Analyzer (Siemens Corporation, Germany) under standard procedures.

### 2.5. Histopathologic Analysis

The paraffin-embedded pancreatic and ileum specimens were sectioned at 4 *μ*m and stained by HE. The morphological study was performed under a light microscope (Olympus Optical Ltd, Tokyo, Japan) by three professional knowledge specialists who were blinded to the experiments. The pancreatic histopathology changes were measured and classified according to the Schmidt score, which included the degree of edema (the extent of diffuse expansion of interlobar septae), inflammatory infiltration (leukocyte infiltration quantity in intralobular or perivascular), hemorrhage, and necrosis (number of foci involved), yielding a maximum score of 16 [16]. The ileum histopathology changes were examined and classified based on Chiu's score with a maximum score of 5 [17]. The scoring criteria in detail are as follows: (1) Grade 0, normal mucosal villi; (2) Grade 1, development of congestion; (3) Grade 2, extension of the subepithelial space with moderate lifting of epithelial layer from the lamina propria; (4) Grade 3, massive epithelial lifting down the sides of villi; (5) Grade 4, denuded villi with lamina propria and dilated capillaries exposed; (6) Grade 5, digestion and disintegration of lamina propria, hemorrhage, and ulceration.

### 2.6. Measurement of Oxidative Stress-Related Enzymes in the Ileum

Ileum extracts were obtained using a commercially extracted buffer (Beyotime, Shanghai, China). After extraction, protein concentrations were determined by the BCA kit. Measurement of the concentration of GSH and MDA and activities of SOD in the ileum were analyzed by kits according to the manufacturer's instructions.

### 2.7. Immunofluorescence and Immunohistochemistry Assay

Sections of immunostaining were consistent with previous methods [18]. Paraffin-embedded tissue sections were put on a stainless shelf and baked in an oven (60°C 1.2 h). Then, slides were subjected to xylene to dewax and hydrated with gradient ethanol for dewaxing. Slides were placed in 10 mM EDTA (pH 9.0) and boiled for 4 min at 121°C in a pressure cooker for antigen retrieval. And then, slides were cooled at room temperature (about 70 min) in the ventilator, immersed in the 10 mM EDTA and washed with proper amount of PBS. Nonspecific sites were blocked via incubation with 10% donkey serum (Jackson Immuno Research, West Grove, USA). The slides were incubated with the following different primary antibodies: rabbit anti-Zo-1 (1 : 200) and rabbit anti-occludin (1 : 200) overnight at 4°C. Then, the sections were incubated with secondary antibody (1 : 200, Abcam, Cambridge, UK) for 1 h in the dark at room temperature. The nuclei were visualized by Fluoroshield Mounting Medium with DAPI (Abcam) staining. Images of the slides were observed and photographed under a fluorescence microscope (Olympus BX63, Tokyo, Japan). Immunofluorescence staining was analyzed using Image ProPlus 6.0 software (Media Cybernetics, Inc., Rockville, MD, USA) for quantitative analysis.

For immunohistochemical staining, after deparaffinization, hydration, antigen retrieval, and serum block, the ileum sections were incubated overnight at 4°C with rabbit anti-NF-*κ*B p65 (1 : 300). Goat anti-rabbit HRP secondary antibody (Maxim Biotech) was added to sections at room temperature. The staining results were visualized using 3,5-diaminobenzidine.

### 2.8. Western Blotting Analysis

Ileum extracts were obtained using a commercially extracted buffer (Beyotime). After extraction, protein concentrations were determined by BCA. Total samples (50 *μ*g) were separated by 8-12% SDS-PAGE, and then, protein was blotted onto a PVDF membrane. After blocking with 5% nonfat milk, blots were incubated with various primary antibodies, followed by HRP-conjugated secondary antibodies and then developed with the use of an ECL reagent. The primary antibodies included Zo-1 (1 : 800), occludin (1 : 1000), RIP3 (1 : 1000), TLR4 (1 : 1000), NF-*κ*B P65 (1 : 1000), and TNF-*α* (1 : 1000), and *β*-actin (1 : 1000) was used as loading control. The protein bands were quantified by Quantity One software (Bio-Rad Laboratories).

### 2.9. Statistical Analysis

The data were expressed by mean ± standard deviation, and data were analyzed using SPSS 20.0 (IBM SPSS, Armonk, NY, USA) statistical software. One-way analysis of variance and the Bonferroni post hoc test were used to determine differences among multiple groups. *P* < 0.05 indicated a statistical difference.

## 3. Results

### 3.1. General Presentations of Rats

After 8 weeks of high-fat diet feeding, HFD-fed rats exhibited significantly higher body weight compared with SRC-fed rats (Figure 1(b)). There was increased adipocyte deposition in the omentum of HFD-fed rats compared with SRC-fed rats (Figure 1(c)).

### 3.2. Serum Activities of AMY and LIP and Serum Levels of TG, TC, DAO, Endotoxin, and Inflammatory Cytokines

**Figure 2:**
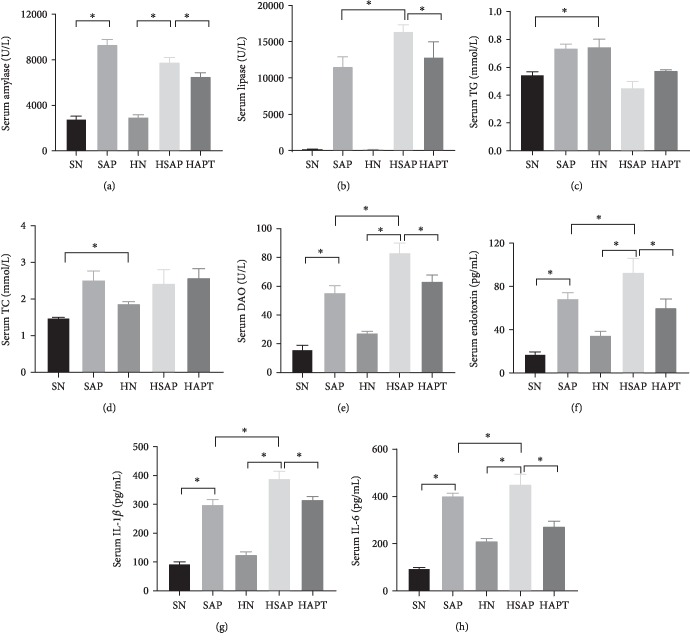
Serum activities of AMY and LIP and serum levels of TG, TC, DAO, serum endotoxin, and inflammatory cytokines. (a, b) Serum activities of AMY and LIP, indicating successful establishment of the SAP model. (c, d) Serum activities of TG and TC, favoring the efficacy of high-fat diet. (e, f) Serum activities of DAO and endotoxin, demonstrating the injury of the intestinal barrier and leakage of DAO and endotoxin into the blood flow. (g, h) High-fat diet and SAP increased serum levels of IL-1 and IL-6.

The serum activities of AMY, LIP, and DAO and the serum levels of endotoxin were markedly higher in the SAP group than in the SN group; meanwhile, higher activities of DAO and serum levels of endotoxin in the HSAP group than in the SAP group could be seen. Levels of serum TG and TC in the HN group were significantly higher than those in the SN groups. Furthermore, compared with the HSAP group, lower activities of DAO and serum levels of endotoxin were detected in the HAPT group (Figures 2(a)–2(f)). The serum levels of IL-6 and IL-1*β* were distinctly higher in the SAP group than in the SN group. The HSAP group demonstrated higher levels of those above inflammatory cytokines compared with the SAP group. In addition, the serum levels of those inflammatory factors in the HAPT group were lower than those in the HSAP group (Figures 2(g) and 2(h)).

### 3.3. Histopathological Analysis of Pancreatic and Ileum Tissues

**Figure 3:**
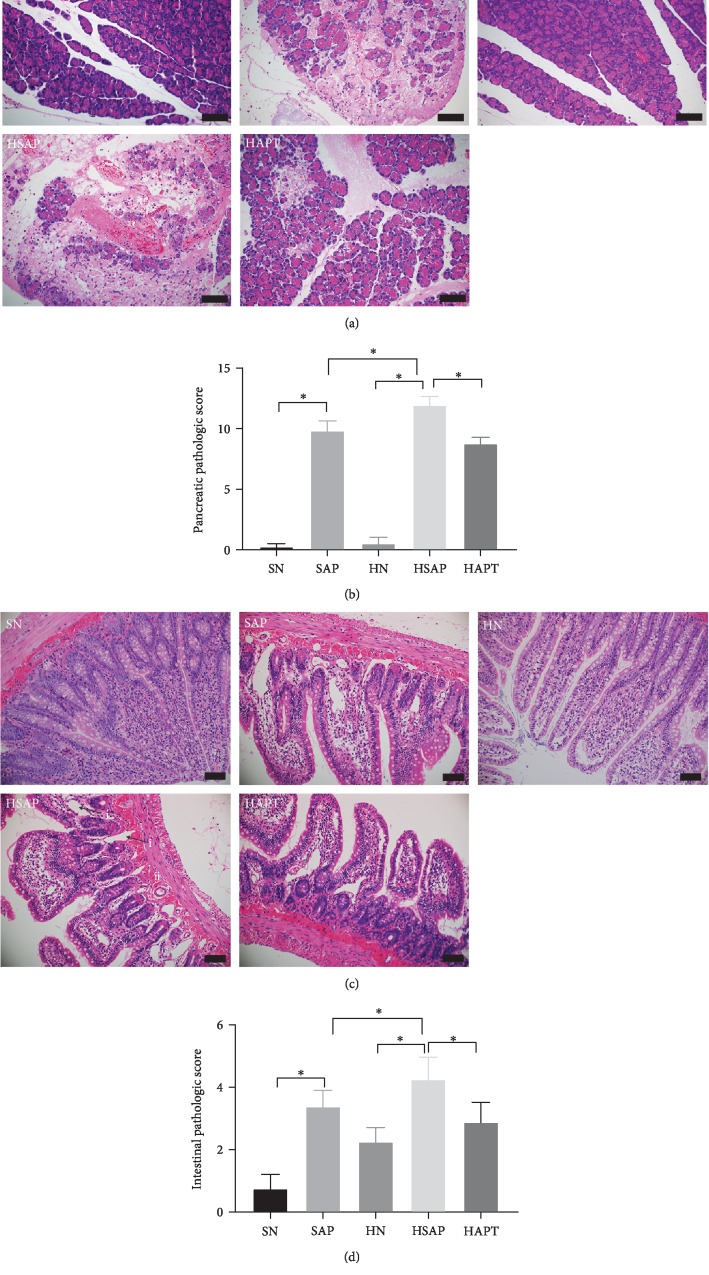
Histological analyses of pancreatic and ileum tissues. (a) Pancreatic H&E staining. Original magnification ×200. (b) Pancreatic pathologic score. (c) Intestinal H&E staining. (i) Showed chyladenectasis and (ii) indicated hemorrhage. Original magnification ×400. (d) Intestinal pathologic score. *P* < 0.05 was considered to be statistically significant.

Edema, hemorrhage, inflammation, and necrosis were observed in the SAP group, the HSAP group, and the HAPT group. The pathological score of the pancreas in the SAP group was markedly higher than that in the SN group, while the HSAP group showed more serious pancreatic injury compared with the SAP group. But the pathological score of the HAPT group was significantly decreased compared with that of the HSAP group (Figures 3(a) and 3(b)).

Edema, hemorrhage, loss in apex of the villus, massive epithelial lifting down the sides of villi, and chyladenectasis were detected in the SAP group, the HSAP group, and the HAPT group. At the same time, edema and loss in apex of the villus can also be observed in the HN group and the HAPT group. The injury score of the ileum in the SAP group was markedly higher than that in the SN group, while the HSAP group exhibited more severe injury compared to the SAP group. However, the HSAP group demonstrated significantly higher pathological score of the ileum compared with the HAPT group (Figures 3(c) and 3(d)).

### 3.4. The Expression of TNF-*α*, NF-*κ*B, RIP3, and TLR4 in the Ileum

**Figure 4:**
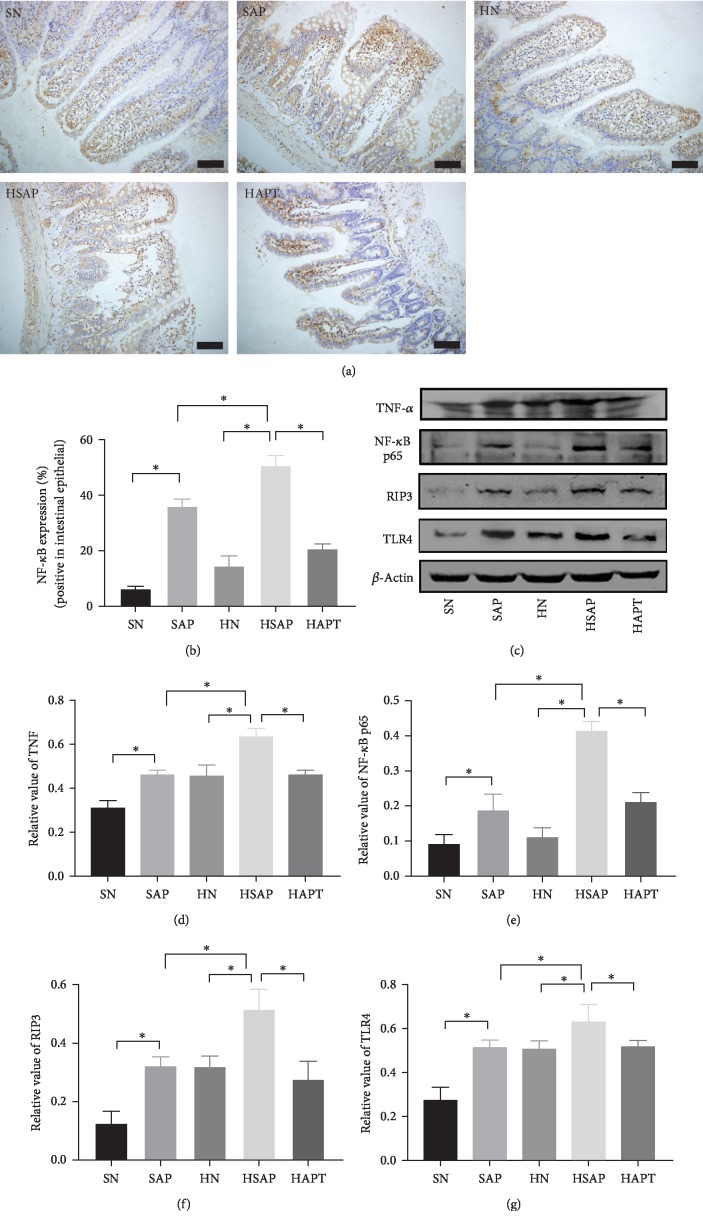
High-fat diet increased inflammatory response to trigger the TLR4-RIP3 pathway. (a) Immunohistochemical staining was performed on ileum sections. Original magnification ×400. (b) The expression of TLR4, TNF-*α*, and RIP3 in the ileum. (d–g) Quantitative analysis of TNF-*α*, NF-*κ*B, RIP3, and TLR4. *P* < 0.05 was considered to be statistically significant.

Expression of TNF-*α* and NF-*κ*B increased in the SAP group and the HSAP group compared with the corresponding SN group and HN group, which indicated inflammatory response inspired in the ileum from systematic inflammation in SAP. Meanwhile, expression of RIP3 was significantly increased in the SAP group than in the SN group, and more in the HSAP group than in the HN group, which demonstrated that maybe necroptosis played an important role in SAP-associated intestinal dysfunction. The HAPT group showed lower expression of TNF-*α* and NF-*κ*B than the HSAP group (Figures 4(a)–4(e)).

Expression of TLR4 increased significantly in the SAP group than in the SN group. Besides, higher expression of TLR4 in the HN group than in the SN group could be seen. In addition, expression of TLR4 was higher in the HSAP group than the SAP group, indicating the role of high-fat diet in intestinal inflammation. Moreover, expression of TLR4 decreased in the HAPT group compared with the HSAP group (Figures 4(c) and 4(g)).

### 3.5. High-Fat Diet and SAP Increased Activities of Oxidative Stress

**Figure 5:**
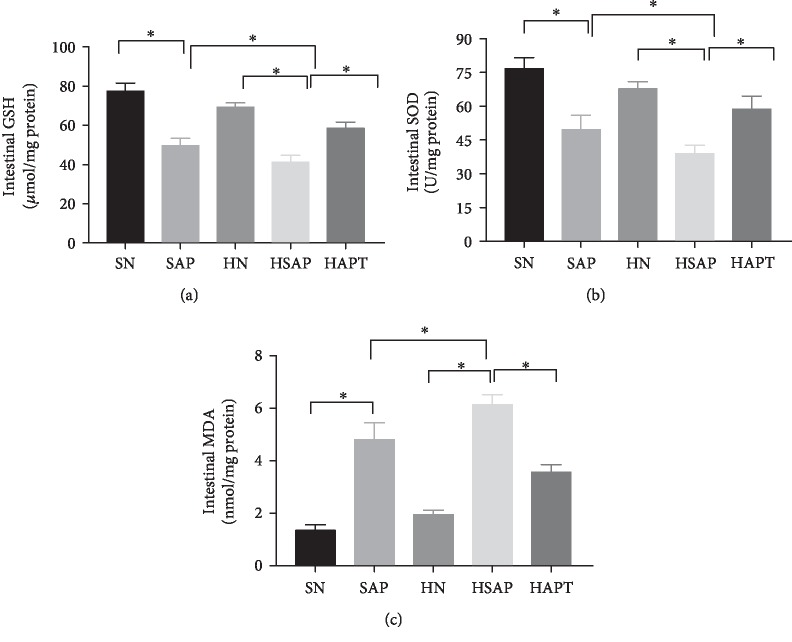
High-fat diet and SAP increased activities of oxidative stress. (a, b) High-fat diet and SAP lower activities of GSH and SOD. (c) High-fat diet and SAP increase activities of MDA. *P* < 0.05 was considered to be statistically significant.

The activities of SOD and levels of GSH were markedly decreased in the SAP group compared with the SN group, ever more reduction was observed in the HSAP group than the HN group as well. Pretreatment with TAK242 increased the activities of SOD and the levels of GSH to some extent, indicating the pivotal role of SAP and HFD in the induction of oxidative stress while TLR4 inhibitors can alleviate this situation. The levels of MDA were significantly increased in the SAP group compared with the SN group, and this tendency was more obvious in the HSAP group compared with the SAP group. After TAK242 pretreatment, the levels of MDA were decreased compared with the HSAP group (Figures 5(a)–5(c)).

### 3.6. High-Fat Diet and SAP Decreased Expression of Zo-1 and Occludin in Ileum Epithelial Cells

**Figure 6:**
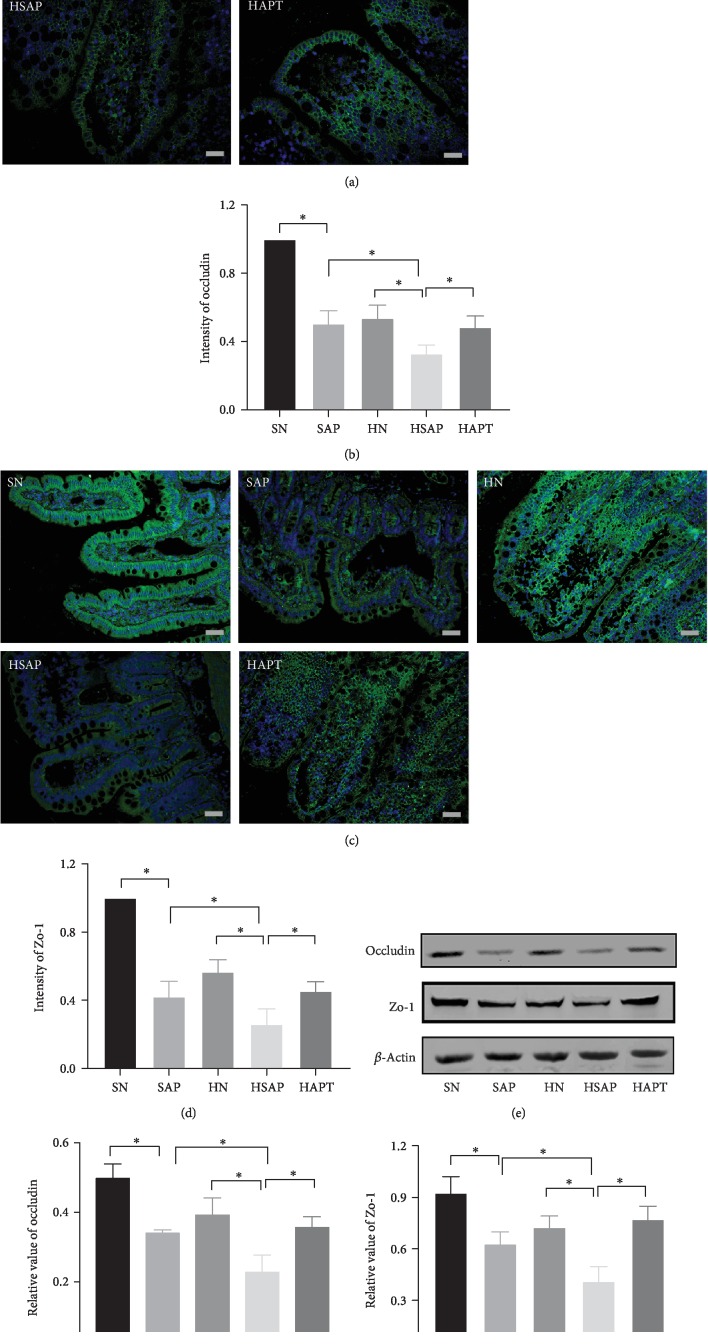
High-fat diet and SAP decreased expression of Zo-1 and occludin in ileum epithelial cells. (a) Immunofluorescence staining (occludin) was performed on ileum sections. Original magnification ×400. (b) Expression of occludin in the ileum. (c) Immunofluorescence staining (Zo-1) was performed on ileum sections. Original magnification ×400. (d) Expression of Zo-1 in the ileum. (e) The expression of tight junction Zo-1 and occludin using western blotting. (f) Quantitative analysis of occludin in the ileum. (g) Quantitative analysis of Zo-1 in the ileum. *P* < 0.05 was considered to be statistically significant.

Expression of Zo-1 and occludin in ileum epithelial cells decreased in the SAP group compared with the SN group, even more reduction was observed in the HSAP group compared with the HN group, indicating the damage of tight junction of intestinal barrier in SAP while high-fat diet aggravated this damage. Besides, after TLR4 inhibitor treatment, the expression of those above two tight junction proteins markedly increased compared with the HSAP group (Figures 6(a)–6(g)).

## 4. Discussion

AP is a common acute abdominal disease with critical complications and high mortality, which is a kind of clinical emergency characterized by edema, bleeding, and necrosis of the pancreas. About 20% of the patients will develop SAP, which can not only cause severe pancreatic injury but can lead to SIRS and even MODS [19]. SIRS and MODS pose a serious threat to the lives of patients with SAP. Researchers indicated that obesity contributed to the high level of oxidative stress increasing the production of proinflammatory factors [20]. In addition, vicious circle was seen in SAP between chronic inflammation in obesity and excessive activation of endoplasmic reticulum stress and oxidative stress [21].

AP is well-known as aseptic inflammation, but there is also chance of infection. Infection is one of the leading causes of severe development and even death [22]. Most of the sources of infection come from the intestinal tract and related to the translocation of intestinal bacteria, the decrease of intestinal barrier function, and the infusion of endotoxin [23]. In SAP, the increase of the intestinal permeability is one of the most important reasons to systemic infection.

In this study, we investigated the differences of the intestinal barrier changes in SAP between HFD-fed rats and SRC-fed rats. In addition, we demonstrated that TLR4 signaling pathway, necroptosis, and oxidative stress, which damage the tight junction among intestinal epithelial cells, play important roles in intestinal barrier dysfunction following SAP on both HFD-fed rats and SRC-fed rats.

Tight junction was formed by four transmembrane proteins including adhesion molecule (junctional adhesion molecules, JAMs), tricellulin, occludin, and claudins, attached cytoplasmic skeleton proteins such as cytoplasmic protein atresia band (Zo) [24]. Zo plays an indispensable role in tight junction and intestinal permeability, and the lack of it leads to the inability to form tight junction [25]. In our study, we found that the expression of Zo-1 and occludin was decreased in the SAP group and the HSAP group associated with the fact that the level of intestinal injury in the HSAP group was higher than the SAP group.

Increased permeability of the small intestine and bacterial translocation were considered as characteristics of pancreatitis-associated gut injury. As an effective biomarker, DAO reflects the integrity and mucosal function of the small intestine. The activities of DAO in the serum may be determined as an approach to assess intestinal barrier function. Besides, there are studies indicating that destruction of the intestinal mucosa mechanical barrier resulted in the release of a large amount of DAO enzymes and active substances [26]. Previous studies have acknowledged that malfunction of the intestinal barrier may result in SIRS and MODS, which may lead to death severely [27, 28]. Our study illustrated decreased width and height of the intestinal villi and tight junctions as well as increased activities of DAO in serum of HFD-fed rats compared with SRC-fed rats following SAP.

There were studies which showed that the intestinal injury in diet-induced nonalcoholic fatty liver disease models could be improved after green tea extract (GTE) treatment which correlated with bile metabolism through lowering TNF-R1, TLR4, and NF-*κ*B expression [29–31]. We assumed that the injury of the intestinal barrier might be correlated with activation of inflammatory response, leading to necroptosis and oxidative stress. Consequently, we intervened the inflammatory response using TLR4 inhibitor, TAK242, to confirm our hypothesis. As expected, pretreatment with TAK242 in HFD rats with SAP improved those above parameters indicating the function of gut barrier. Considering the above results, we speculated that inhibiting the TLR4 signal pathway plays a positive role in protecting intestinal barrier function associated with SAP.

As we all know, proinflammatory cytokines, such as IL-1*β*, IL-6, and TNF-*α*, play an essential role in the pathogenesis of SAP through originating, magnifying, and perpetuating the inflammatory response in SAP. What is more, TNF-*α* is the earliest and primary endogenous mediator in the inflammatory response [32], which activates and amplifies the inflammatory cascade, triggering necroptosis through the classical pathway [33]. In our study, high-fat diet-fed rats with SAP showed more severe inflammatory response and increased level of necroptosis and oxidative stress compared with the SAP group. It is suspected that inflammation and higher level of oxidative stress following SAP posed fat rats to produce excessive inflammatory factors to protect themselves.

Here, we showed that HFD was associated with higher level of oxidative stress, SAP contributed to increased oxidative stress as well. From our studies, SAP with HFD demonstrated the highest levels of MDA, lowest activities of SOD, and lowest levels of GSH compared with other groups, all of which indicated that HFD and oxidative stress might take part in the dysfunction of gut barrier. Using TLR4 inhibitor, injury of the intestine was improved. It came to our mind that inhibiting inflammation and improving hyperlipidemia may be more useful in the prognosis of SAP.

Programed cell death plays an important role in multiple physiological processes, such as cell growth, reproduction, and differentiation. Decades ago, apoptosis was considered as the only form of programed cell death, while necrosis was considered as an unregulated cell death [34]. Recent acknowledged necroptosis has been determined to be regulated by the tumor necrosis factor receptor (TNFR) family, receptor-interacting protein 1 (RIP1), receptor-interacting protein 3 (RIP3), and mixed lineage kinase-like (MLKL) proteins. There were studies indicating that inhibition of programmed necrosis alleviated the severity of AP [35, 36]. In our study, we demonstrated a positive correlation between pathological score and levels of necroptosis while a negative correlation between pancreatic injury and expression of tight junction proteins was also demonstrated. Moreover, inhibition of necroptosis improved the dysfunction of intestinal barrier associated with AP more apparently in high-fat diet-fed rat group. TLR4 inhibitor pretreatment significantly reduced the degree of intestinal necroptosis as well as production of intestinal proinflammatory cytokines via regulating the TLR4 inflammatory signaling pathway and thus protected intestinal integrity and function in rats following sodium taurocholate challenge. Consistent with previous studies, inhibiting RIP3-mediated IEC necroptosis preserves epithelial barrier integrity and antibacterial defense, maintains homeostasis, and prevents chronic intestinal inflammation [37]. Furthermore, we observed higher level of necroptosis and increased production of proinflammatory cytokines in high-fat diet-fed rats following SAP compared with standard-fed group.

Taken together, pancreatitis-associated intestinal barrier dysfunction was found in the SAP group and more severely in high-fat diet-fed rats than standard-fed rats. Inhibition of TLR4 significantly reduced the severity of intestinal barrier malfunction owing to a reduction of the systemic concentration of cytokines and decreasing level of necroptosis by downregulating RIP3 expression. Consequently, treatment with TAK242 improved SAP-induced intestinal barrier injury, making it possible for TAK242 as a novel and adjuvant therapy to treat intestinal barrier injury with SAP, especially in those patients with obesity.

## 5. Conclusion

High-fat diet aggravated inflammatory reactions, necroptosis, and oxidative stress to increase SAP-induced intestinal barrier injury. TAK242 could reduce the inflammatory reaction, alleviate necroptosis, and lower the level of oxidative stress, protecting the intestinal barrier dysfunction from SAP in high-fat diet-fed rats. An effective method to reduce obesity and alleviate inflammation still has not been illustrated and needed further research.

## Data Availability

The data used to support the findings of this study are included within the article.
